# American International Congress on Tuberculosis

**Published:** 1904-02

**Authors:** 


Secretary.

### American International Congress on Tuberculosis.

To be held October 3, 4 and 5, 1904, under the auspices of the Universal Exposition, at St. Louis, 1904, and of the American Congress on Tuberculosis.

HONORARY PRESIDENTS.

*Lay.*—Hon. John Hay, Hon. Gen. Russell A. Alger, Hon. Ex-

Judge A. H. Dailey, Hon. Judge C. G. Garrison, Hon. Stephen B. Elkins.

*Medical.*—Prof. Dr. M. Benedikt, Dr. A. N. Bell, Prof. Dr. Chas. H. Hughes, Gen. Presley M. Rixey, M. D., Gen. Nicholas Senn, M. D.

*Council.*—Moritz Ellinger, Esq., Chairman; J. Mount Bleyer, M. D., New York City; A. P. Grinnell, M. D., Vermont; H. Edwin Lewis, M. D., Vermont; Richard J. Nunn, M. D., Georgia; W. F. Drewry, M. D., Virginia; M. K. Kassabian, M. D., Pennsylvania; J. W. P. Smithwick, M. D., North Carolina.

*Officers.*—President, E. J. Barrick, M. D., Toronto, Ontario; First Vice-President, F. E. Daniel, M. D., Austin, Texas; Second Vice-President, Ex-Chief Justice, L. Bradford Prince, Santa Fe, New Mexico; Third Vice-President, Dr. Charles K. Cole, Helena, Montana; Fourth Vice-President, Dr. Sofus F. Nelson, Pulman, Washington; Fifth Vice-President, Dr. A. M. Linn, Des Moines, Iowa; Secretary, Samuel Bell Thomas, 116 Nassau Street, New York; Treasurer, Clark Bell, 39 Broadway, New York.

New York, September 21, 1903.

*To the Officers, Delegates and Members of the American Congress on Tuberculosis:*

It affords the executive officers of the American Congress on Tuberculosis great pleasure to announce the reception of the following letter from the government of the United States, Department of State:

"Department of State,
Washington, August 29, 1903.

*Clark Bell, Esq., Chairman of the Executive Committee of the American Congress on Tuberculosis, 39 Broadway, New York.*

Sir: Referring to the correspondence which the department has recently had with you concerning the desire of the Committee on Organization of the proposed American Congress on Tuberculosis to be held at St. Louis, in October, 1904, to have this government give its support to the invitation which the committee has addressed to each American government to be represented at the congress, I enclose herewith a draft of an instruction to each diplomatic representative of the United States in the Western Hemisphere. The department will be pleased to consider any changes in, or additions to the draft, you may suggest. I am, sir,

Your obedient servant,
F. B. Loomis,
Assistant Secretary."

LETTER OF INSTRUCTION TO CONSULS.

"DEPARTMENT OF STATE,
WASHINGTON, D. C.

SIR: The department is informed by Mr. Howard J. Rogers, Director of International Congresses of the Universal Exposition to be held in St. Louis in 1904, that the American Congress on Tuberculosis has been placed on its list of official congresses and that the dates for said congresses will be October 3, 4 and 5, 1904.

The department is also advised by Mr. Clark Bell, Chairman of the Committee of Organization of the Congress, that the Executive Committee and Officers of the Congress have sent to the government of each American country an invitation for official representation by that government, in the congress; and the request is made of the department to give such support to the invitation as it properly may.

The humanitarian object which this congress has in view to reach, by the discussion of scientific men, some result in arresting the spread and averting, so far as it may be found possible, the ravages of this dreadful disease which now falls with such terrible force and fatality upon the people of the Western Hemisphere, can not but enlist the sympathy and approval of the government to which you are accredited.

The department will, therefore, be pleased to have you say to that government that this government is in entire sympathy with the work of the proposed congress, and would be pleased to learn that the government of ——————— took a like interest in its success by the acceptance of the committee's invitation and the appointment of three or more scientific gentlemen to represent it at the congress.

This government would also be pleased if that of ———————— could find it convenient to comply with the request of the committee to give the matter publicity in order that it may come to the knowledge of interested organizations and public spirited citizens of that country. I am, Sir,

Your obedient servant,

Secretary of State."

This splendid expression of the sympathy of the government of the United States insures a cordial reception of our work in the nations of the Western Hemisphere. It evinces and illustrates the paternal policy of our government in aiding every effort for the protection of the health and the lives of our people when menaced from any form of disease that science has found to be communicable and preventible.

The above instructions were by the Department of State sent to Ambassador Choate. The instructions to France, Denmark and the Netherlands and in the same language; *mutatis mutantis.—Advanced Sheets Medico-Legal Journal, N. Y.*

